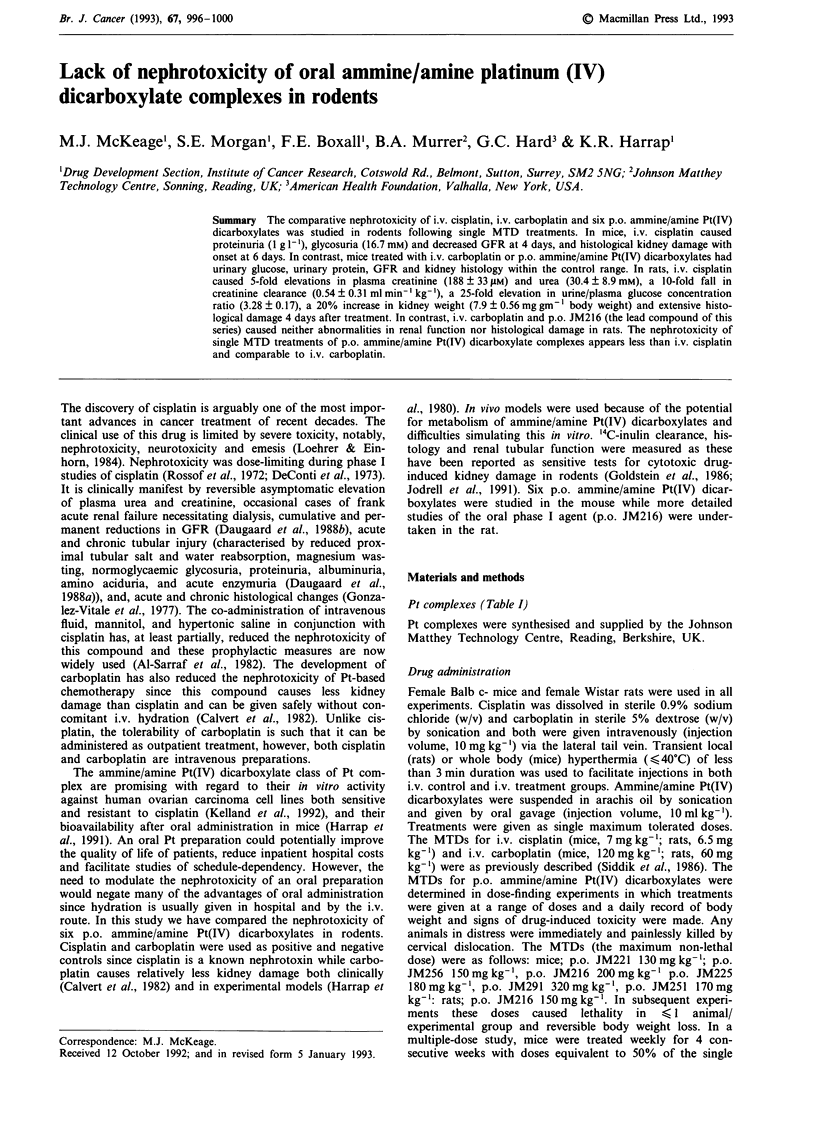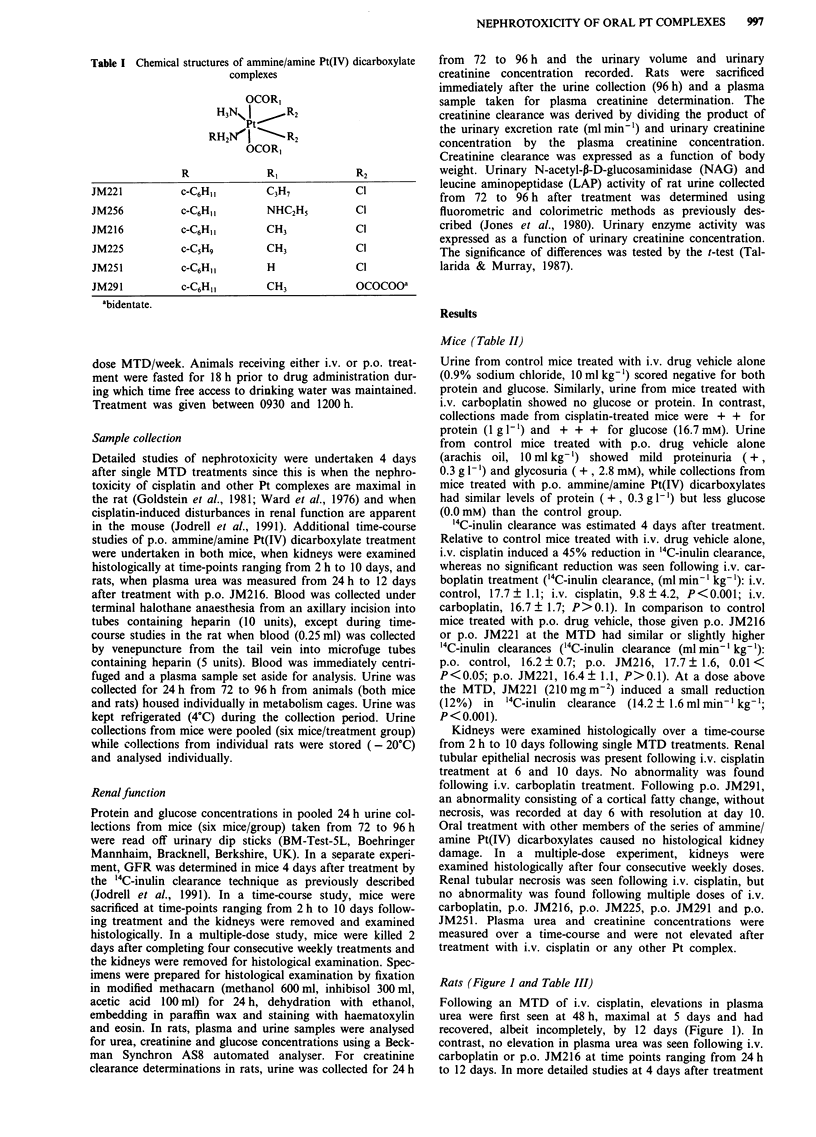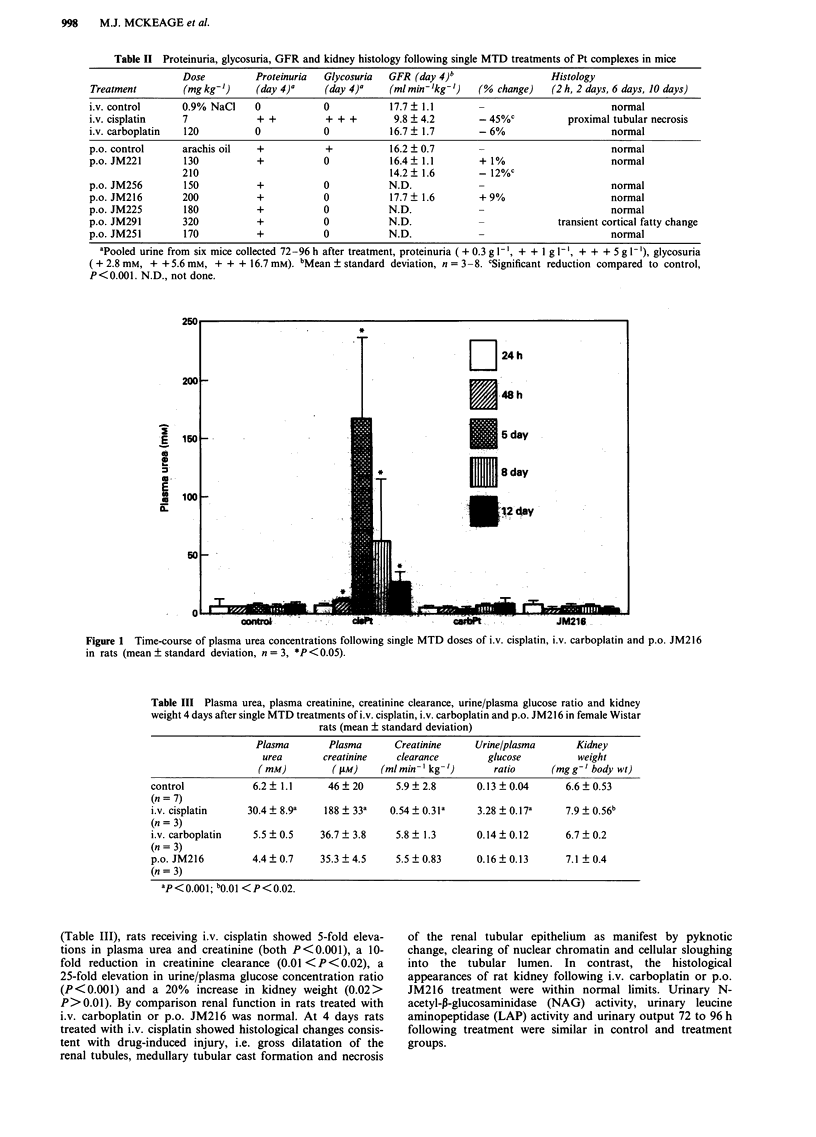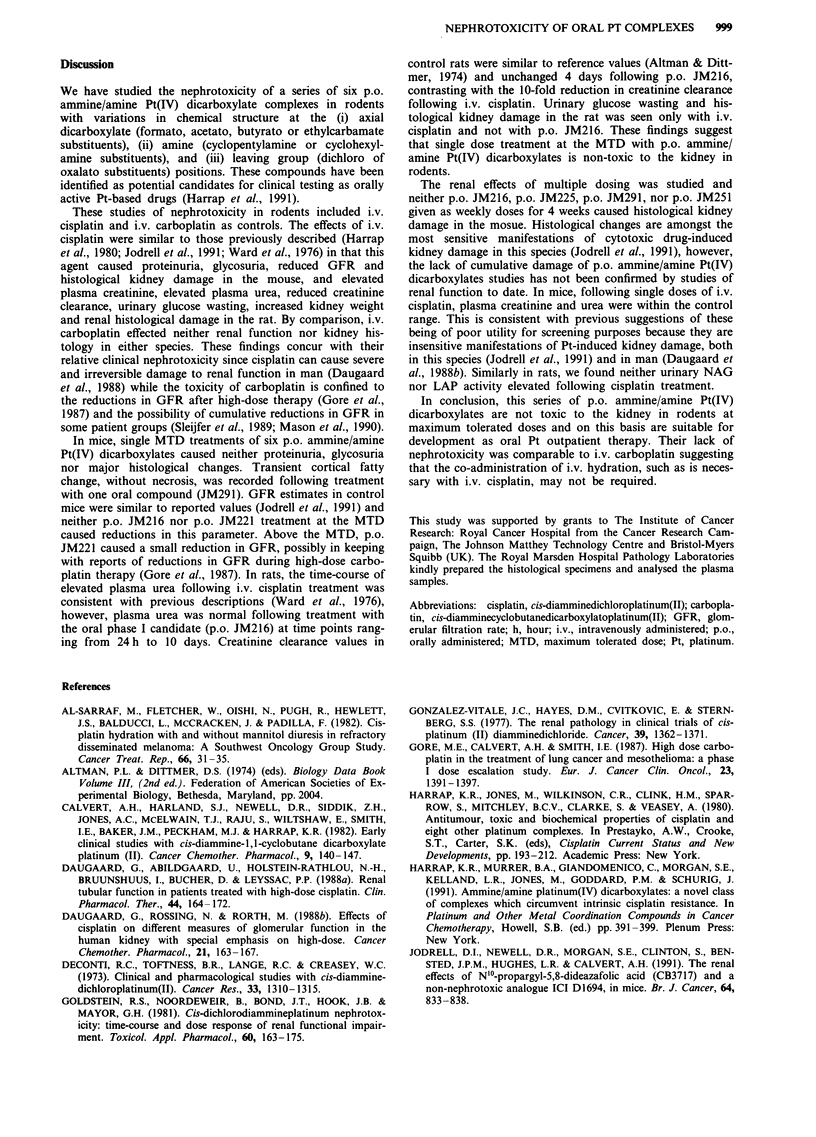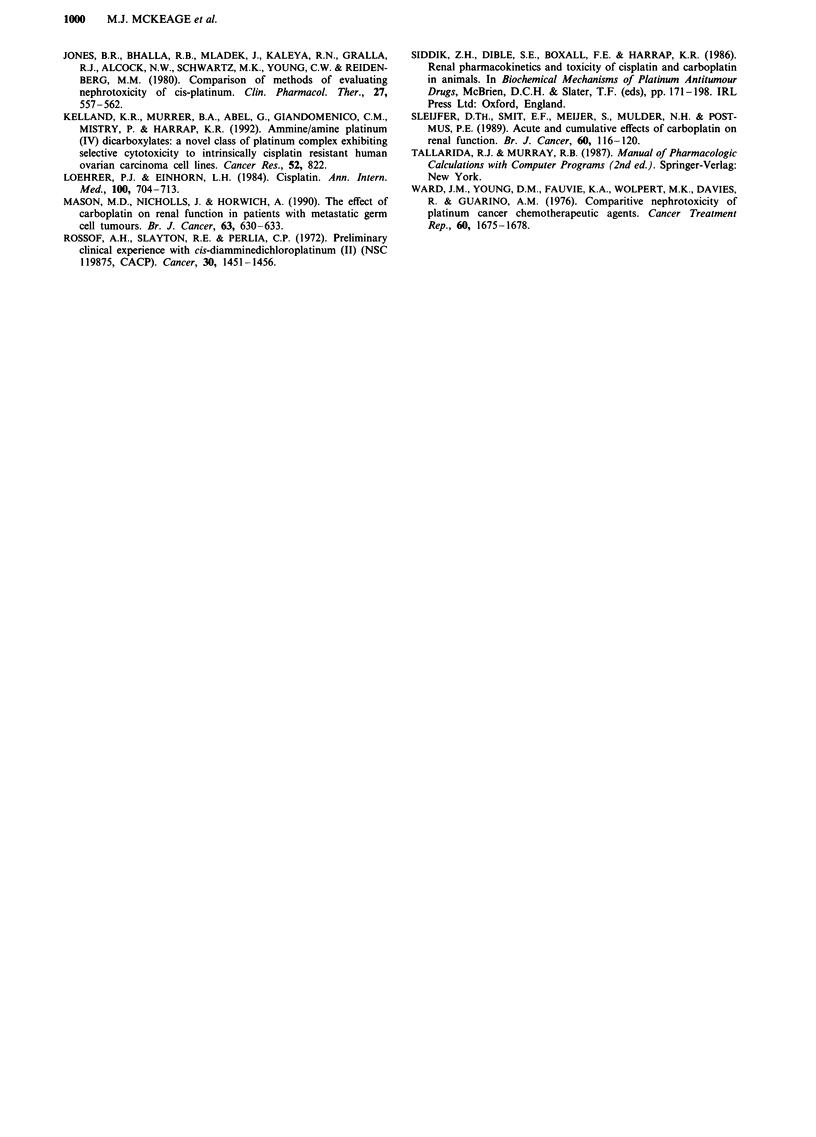# Lack of nephrotoxicity of oral ammine/amine platinum (IV) dicarboxylate complexes in rodents.

**DOI:** 10.1038/bjc.1993.182

**Published:** 1993-05

**Authors:** M. J. McKeage, S. E. Morgan, F. E. Boxall, B. A. Murrer, G. C. Hard, K. R. Harrap

**Affiliations:** Drug Development Section, Institute of Cancer Research, Sutton, Surrey.

## Abstract

The comparative nephrotoxicity of i.v. cisplatin, i.v. carboplatin and six p.o. ammine/amine Pt(IV) dicarboxylates was studied in rodents following single MTD treatments. In mice, i.v. cisplatin caused proteinuria (1 g l-1), glycosuria (16.7 mM) and decreased GFR at 4 days, and histological kidney damage with onset at 6 days. In contrast, mice treated with i.v. carboplatin or p.o. ammine/amine Pt(IV) dicarboxylates had urinary glucose, urinary protein, GFR and kidney histology within the control range. In rats, i.v. cisplatin caused 5-fold elevations in plasma creatinine (188 +/- 33 microM) and urea (30.4 +/- 8.9 mM), a 10-fold fall in creatinine clearance (0.54 +/- 0.31 ml min-1 kg-1), a 25-fold elevation in urine/plasma glucose concentration ratio (3.28 +/- 0.17), a 20% increase in kidney weight (7.9 +/- 0.56 mg gm-1 body weight) and extensive histological damage 4 days after treatment. In contrast, i.v. carboplatin and p.o. JM216 (the lead compound of this series) caused neither abnormalities in renal function nor histological damage in rats. The nephrotoxicity of single MTD treatments of p.o. ammine/amine Pt(IV) dicarboxylate complexes appears less than i.v. cisplatin and comparable to i.v. carboplatin.


Br. J. Cancer (1993), 67, 996–1000 © Macmillan Press Ltd., 1993

### Lack of nephrotoxicity of oral ammine/amine platinum (IV) dicarboxylate complexes in rodents

M.J. McKeage[1], S.E. Morgan[1], F.E. Boxall[1], B.A. Murrer[2], G.C. Hard[3] & K.R. Harrap[1]

[1]*Drug Development Section, Institute of Cancer Research, Cotswold Rd., Belmont, Sutton, Surrey, SM2 5NG;* [2]*Johnson Matthey Technology Centre, Sonning, Reading, UK;* [3]*American Health Foundation, Valhalla, New York, USA.*

**Summary** The comparative nephrotoxicity of i.v. cisplatin, i.v. carboplatin and six p.o. ammine/amine Pt(IV) dicarboxylates was studied in rodents following single MTD treatments. In mice, i.v. cisplatin caused proteinuria (1 g $l^{-1}$), glycosuria (16.7 mM) and decreased GFR at 4 days, and histological kidney damage with onset at 6 days. In contrast, mice treated with i.v. carboplatin or p.o. ammine/amine Pt(IV) dicarboxylates had urinary glucose, urinary protein, GFR and kidney histology within the control range. In rats, i.v. cisplatin caused 5-fold elevations in plasma creatinine ($188 \pm 33$ μM) and urea ($30.4 \pm 8.9$ mM), a 10-fold fall in creatinine clearance ($0.54 \pm 0.31$ ml $min^{-1}$ $kg^{-1}$), a 25-fold elevation in urine/plasma glucose concentration ratio ($3.28 \pm 0.17$), a 20% increase in kidney weight ($7.9 \pm 0.56$ mg $gm^{-1}$ body weight) and extensive histological damage 4 days after treatment. In contrast, i.v. carboplatin and p.o. JM216 (the lead compound of this series) caused neither abnormalities in renal function nor histological damage in rats. The nephrotoxicity of single MTD treatments of p.o. ammine/amine Pt(IV) dicarboxylate complexes appears less than i.v. cisplatin and comparable to i.v. carboplatin.

The discovery of cisplatin is arguably one of the most important advances in cancer treatment of recent decades. The clinical use of this drug is limited by severe toxicity, notably, nephrotoxicity, neurotoxicity and emesis (Loehrer & Einhorn, 1984). Nephrotoxicity was dose-limiting during phase I studies of cisplatin (Rossof *et al.*, 1972; DeConti *et al.*, 1973). It is clinically manifest by reversible asymptomatic elevation of plasma urea and creatinine, occasional cases of frank acute renal failure necessitating dialysis, cumulative and permanent reductions in GFR (Daugaard *et al.*, 1988*b*), acute and chronic tubular injury (characterised by reduced proximal tubular salt and water reabsorption, magnesium wasting, normoglycaemic glycosuria, proteinuria, albuminuria, amino aciduria, and acute enzymuria (Daugaard *et al.*, 1988*a*)), and, acute and chronic histological changes (Gonzalez-Vitale *et al.*, 1977). The co-administration of intravenous fluid, mannitol, and hypertonic saline in conjunction with cisplatin has, at least partially, reduced the nephrotoxicity of this compound and these prophylactic measures are now widely used (Al-Sarraf *et al.*, 1982). The development of carboplatin has also reduced the nephrotoxicity of Pt-based chemotherapy since this compound causes less kidney damage than cisplatin and can be given safely without concomitant i.v. hydration (Calvert *et al.*, 1982). Unlike cisplatin, the tolerability of carboplatin is such that it can be administered as outpatient treatment, however, both cisplatin and carboplatin are intravenous preparations.

The ammine/amine Pt(IV) dicarboxylate class of Pt complex are promising with regard to their *in vitro* activity against human ovarian carcinoma cell lines both sensitive and resistant to cisplatin (Kelland *et al.*, 1992), and their bioavailability after oral administration in mice (Harrap *et al.*, 1991). An oral Pt preparation could potentially improve the quality of life of patients, reduce inpatient hospital costs and facilitate studies of schedule-dependency. However, the need to modulate the nephrotoxicity of an oral preparation would negate many of the advantages of oral administration since hydration is usually given in hospital and by the i.v. route. In this study we have compared the nephrotoxicity of six p.o. ammine/amine Pt(IV) dicarboxylates in rodents. Cisplatin and carboplatin were used as positive and negative controls since cisplatin is a known nephrotoxin while carboplatin causes relatively less kidney damage both clinically (Calvert *et al.*, 1982) and in experimental models (Harrap *et al.*, 1980). *In vivo* models were used because of the potential for metabolism of ammine/amine Pt(IV) dicarboxylates and difficulties simulating this *in vitro*. $^{14}$C-inulin clearance, histology and renal tubular function were measured as these have been reported as sensitive tests for cytotoxic drug-induced kidney damage in rodents (Goldstein *et al.*, 1986; Jodrell *et al.*, 1991). Six p.o. ammine/amine Pt(IV) dicarboxylates were studied in the mouse while more detailed studies of the oral phase I agent (p.o. JM216) were undertaken in the rat.

Correspondence: M.J. McKeage.
Received 12 October 1992; and in revised form 5 January 1993.

### Materials and methods

### *Pt complexes (Table I)*

Pt complexes were synthesised and supplied by the Johnson Matthey Technology Centre, Reading, Berkshire, UK.

### *Drug administration*

Female Balb c- mice and female Wistar rats were used in all experiments. Cisplatin was dissolved in sterile 0.9% sodium chloride (w/v) and carboplatin in sterile 5% dextrose (w/v) by sonication and both were given intravenously (injection volume, 10 mg $kg^{-1}$) via the lateral tail vein. Transient local (rats) or whole body (mice) hyperthermia ($\leqslant$40°C) of less than 3 min duration was used to facilitate injections in both i.v. control and i.v. treatment groups. Ammine/amine Pt(IV) dicarboxylates were suspended in arachis oil by sonication and given by oral gavage (injection volume, 10 ml $kg^{-1}$). Treatments were given as single maximum tolerated doses. The MTDs for i.v. cisplatin (mice, 7 mg $kg^{-1}$; rats, 6.5 mg $kg^{-1}$) and i.v. carboplatin (mice, 120 mg $kg^{-1}$; rats, 60 mg $kg^{-1}$) were as previously described (Siddik *et al.*, 1986). The MTDs for p.o. ammine/amine Pt(IV) dicarboxylates were determined in dose-finding experiments in which treatments were given at a range of doses and a daily record of body weight and signs of drug-induced toxicity were made. Any animals in distress were immediately and painlessly killed by cervical dislocation. The MTDs (the maximum non-lethal dose) were as follows: mice; p.o. JM221 130 mg $kg^{-1}$; p.o. JM256 150 mg $kg^{-1}$, p.o. JM216 200 mg $kg^{-1}$ p.o. JM225 180 mg $kg^{-1}$, p.o. JM291 320 mg $kg^{-1}$, p.o. JM251 170 mg $kg^{-1}$: rats; p.o. JM216 150 mg $kg^{-1}$. In subsequent experiments these doses caused lethality in $\leqslant 1$ animal/experimental group and reversible body weight loss. In a multiple-dose study, mice were treated weekly for 4 consecutive weeks with doses equivalent to 50% of the single

**Table I** Chemical structures of ammine/amine Pt(IV) dicarboxylate complexes

| | R | $R_1$ | $R_2$ |
|---|---|---|---|
| JM221 | c-$C_6H_{11}$ | $C_3H_7$ | Cl |
| JM256 | c-$C_6H_{11}$ | $NHC_2H_5$ | Cl |
| JM216 | c-$C_6H_{11}$ | $CH_3$ | Cl |
| JM225 | c-$C_5H_9$ | $CH_3$ | Cl |
| JM251 | c-$C_6H_{11}$ | H | Cl |
| JM291 | c-$C_6H_{11}$ | $CH_3$ | OCOCOO[a] |

[a]bidentate.

dose MTD/week. Animals receiving either i.v. or p.o. treatment were fasted for 18 h prior to drug administration during which time free access to drinking water was maintained. Treatment was given between 0930 and 1200 h.

*Sample collection*

Detailed studies of nephrotoxicity were undertaken 4 days after single MTD treatments since this is when the nephrotoxicity of cisplatin and other Pt complexes are maximal in the rat (Goldstein *et al.*, 1981; Ward *et al.*, 1976) and when cisplatin-induced disturbances in renal function are apparent in the mouse (Jodrell *et al.*, 1991). Additional time-course studies of p.o. ammine/amine Pt(IV) dicarboxylate treatment were undertaken in both mice, when kidneys were examined histologically at time-points ranging from 2 h to 10 days, and rats, when plasma urea was measured from 24 h to 12 days after treatment with p.o. JM216. Blood was collected under terminal halothane anaesthesia from an axillary incision into tubes containing heparin (10 units), except during time-course studies in the rat when blood (0.25 ml) was collected by venepuncture from the tail vein into microfuge tubes containing heparin (5 units). Blood was immediately centrifuged and a plasma sample set aside for analysis. Urine was collected for 24 h from 72 to 96 h from animals (both mice and rats) housed individually in metabolism cages. Urine was kept refrigerated (4°C) during the collection period. Urine collections from mice were pooled (six mice/treatment group) while collections from individual rats were stored ( – 20°C) and analysed individually.

*Renal function*

Protein and glucose concentrations in pooled 24 h urine collections from mice (six mice/group) taken from 72 to 96 h were read off urinary dip sticks (BM-Test-5L, Boehringer Mannhaim, Bracknell, Berkshire, UK). In a separate experiment, GFR was determined in mice 4 days after treatment by the $^{14}$C-inulin clearance technique as previously described (Jodrell *et al.*, 1991). In a time-course study, mice were sacrificed at time-points ranging from 2 h to 10 days following treatment and the kidneys were removed and examined histologically. In a multiple-dose study, mice were killed 2 days after completing four consecutive weekly treatments and the kidneys were removed for histological examination. Specimens were prepared for histological examination by fixation in modified methacarn (methanol 600 ml, inhibisol 300 ml, acetic acid 100 ml) for 24 h, dehydration with ethanol, embedding in paraffin wax and staining with haematoxylin and eosin. In rats, plasma and urine samples were analysed for urea, creatinine and glucose concentrations using a Beckman Synchron AS8 automated analyser. For creatinine clearance determinations in rats, urine was collected for 24 h from 72 to 96 h and the urinary volume and urinary creatinine concentration recorded. Rats were sacrificed immediately after the urine collection (96 h) and a plasma sample taken for plasma creatinine determination. The creatinine clearance was derived by dividing the product of the urinary excretion rate (ml min$^{-1}$) and urinary creatinine concentration by the plasma creatinine concentration. Creatinine clearance was expressed as a function of body weight. Urinary N-acetyl-β-D-glucosaminidase (NAG) and leucine aminopeptidase (LAP) activity of rat urine collected from 72 to 96 h after treatment was determined using fluorometric and colorimetric methods as previously described (Jones *et al.*, 1980). Urinary enzyme activity was expressed as a function of urinary creatinine concentration. The significance of differences was tested by the *t*-test (Tallarida & Murray, 1987).

### Results

*Mice (Table II)*

Urine from control mice treated with i.v. drug vehicle alone (0.9% sodium chloride, 10 ml kg$^{-1}$) scored negative for both protein and glucose. Similarly, urine from mice treated with i.v. carboplatin showed no glucose or protein. In contrast, collections made from cisplatin-treated mice were + + for protein (1 g l$^{-1}$) and + + + for glucose (16.7 mM). Urine from control mice treated with p.o. drug vehicle alone (arachis oil, 10 ml kg$^{-1}$) showed mild proteinuria ( +, 0.3 g l$^{-1}$) and glycosuria ( + , 2.8 mM), while collections from mice treated with p.o. ammine/amine Pt(IV) dicarboxylates had similar levels of protein ( + , 0.3 g l$^{-1}$) but less glucose (0.0 mM) than the control group.

$^{14}$C-inulin clearance was estimated 4 days after treatment. Relative to control mice treated with i.v. drug vehicle alone, i.v. cisplatin induced a 45% reduction in $^{14}$C-inulin clearance, whereas no significant reduction was seen following i.v. carboplatin treatment ($^{14}$C-inulin clearance, (ml min$^{-1}$ kg$^{-1}$): i.v. control, 17.7 ± 1.1; i.v. cisplatin, 9.8 ± 4.2, $P<0.001$; i.v. carboplatin, 16.7 ± 1.7; $P>0.1$). In comparison to control mice treated with p.o. drug vehicle, those given p.o. JM216 or p.o. JM221 at the MTD had similar or slightly higher $^{14}$C-inulin clearances ($^{14}$C-inulin clearance (ml min$^{-1}$ kg$^{-1}$): p.o. control, 16.2 ± 0.7; p.o. JM216, 17.7 ± 1.6, $0.01 < P < 0.05$; p.o. JM221, 16.4 ± 1.1, $P>0.1$). At a dose above the MTD, JM221 (210 mg m$^{-2}$) induced a small reduction (12%) in $^{14}$C-inulin clearance (14.2 ± 1.6 ml min$^{-1}$ kg$^{-1}$; $P<0.001$).

Kidneys were examined histologically over a time-course from 2 h to 10 days following single MTD treatments. Renal tubular epithelial necrosis was present following i.v. cisplatin treatment at 6 and 10 days. No abnormality was found following i.v. carboplatin treatment. Following p.o. JM291, an abnormality consisting of a cortical fatty change, without necrosis, was recorded at day 6 with resolution at day 10. Oral treatment with other members of the series of ammine/amine Pt(IV) dicarboxylates caused no histological kidney damage. In a multiple-dose experiment, kidneys were examined histologically after four consecutive weekly doses. Renal tubular necrosis was seen following i.v. cisplatin, but no abnormality was found following multiple doses of i.v. carboplatin, p.o. JM216, p.o. JM225, p.o. JM291 and p.o. JM251. Plasma urea and creatinine concentrations were measured over a time-course and were not elevated after treatment with i.v. cisplatin or any other Pt complex.

*Rats (Figure 1 and Table III)*

Following an MTD of i.v. cisplatin, elevations in plasma urea were first seen at 48 h, maximal at 5 days and had recovered, albeit incompletely, by 12 days (Figure 1). In contrast, no elevation in plasma urea was seen following i.v. carboplatin or p.o. JM216 at time points ranging from 24 h to 12 days. In more detailed studies at 4 days after treatment

**Table II** Proteinuria, glycosuria, GFR and kidney histology following single MTD treatments of Pt complexes in mice

| *Treatment* | *Dose (mg kg⁻¹)* | *Proteinuria (day 4)[a]* | *Glycosuria (day 4)[a]* | *GFR (day 4)[b] (ml min⁻¹kg⁻¹)* | *(% change)* | *Histology (2 h, 2 days, 6 days, 10 days)* |
|---|---|---|---|---|---|---|
| i.v. control | 0.9% NaCl | 0 | 0 | 17.7 ± 1.1 | – | normal |
| i.v. cisplatin | 7 | + + | + + + | 9.8 ± 4.2 | – 45%[c] | proximal tubular necrosis |
| i.v. carboplatin | 120 | 0 | 0 | 16.7 ± 1.7 | – 6% | normal |
| p.o. control | arachis oil | + | + | 16.2 ± 0.7 | – | normal |
| p.o. JM221 | 130 | + | 0 | 16.4 ± 1.1 | + 1% | normal |
| | 210 | | | 14.2 ± 1.6 | – 12%[c] | |
| p.o. JM256 | 150 | + | 0 | N.D. | – | normal |
| p.o. JM216 | 200 | + | 0 | 17.7 ± 1.6 | + 9% | normal |
| p.o. JM225 | 180 | + | 0 | N.D. | – | normal |
| p.o. JM291 | 320 | + | 0 | N.D. | – | transient cortical fatty change |
| p.o. JM251 | 170 | + | 0 | N.D. | – | normal |

[a]Pooled urine from six mice collected 72–96 h after treatment, proteinuria ( + 0.3 g l⁻¹, + + 1 g l⁻¹, + + + 5 g l⁻¹), glycosuria ( + 2.8 mM, + + 5.6 mM, + + + 16.7 mM). [b]Mean ± standard deviation, $n = 3–8$. [c]Significant reduction compared to control, $P < 0.001$. N.D., not done.

**Figure 1** Time-course of plasma urea concentrations following single MTD doses of i.v. cisplatin, i.v. carboplatin and p.o. JM216 in rats (mean ± standard deviation, $n = 3$, $^*P < 0.05$).

**Table III** Plasma urea, plasma creatinine, creatinine clearance, urine/plasma glucose ratio and kidney weight 4 days after single MTD treatments of i.v. cisplatin, i.v. carboplatin and p.o. JM216 in female Wistar rats (mean ± standard deviation)

| | *Plasma urea (mM)* | *Plasma creatinine (μM)* | *Creatinine clearance (ml min⁻¹ kg⁻¹)* | *Urine/plasma glucose ratio* | *Kidney weight (mg g⁻¹ body wt)* |
|---|---|---|---|---|---|
| control ($n = 7$) | 6.2 ± 1.1 | 46 ± 20 | 5.9 ± 2.8 | 0.13 ± 0.04 | 6.6 ± 0.53 |
| i.v. cisplatin ($n = 3$) | 30.4 ± 8.9[a] | 188 ± 33[a] | 0.54 ± 0.31[a] | 3.28 ± 0.17[a] | 7.9 ± 0.56[b] |
| i.v. carboplatin ($n = 3$) | 5.5 ± 0.5 | 36.7 ± 3.8 | 5.8 ± 1.3 | 0.14 ± 0.12 | 6.7 ± 0.2 |
| p.o. JM216 ($n = 3$) | 4.4 ± 0.7 | 35.3 ± 4.5 | 5.5 ± 0.83 | 0.16 ± 0.13 | 7.1 ± 0.4 |

[a]$P < 0.001$; [b]$0.01 < P < 0.02$.

(Table III), rats receiving i.v. cisplatin showed 5-fold elevations in plasma urea and creatinine (both $P < 0.001$), a 10-fold reduction in creatinine clearance ($0.01 < P < 0.02$), a 25-fold elevation in urine/plasma glucose concentration ratio ($P < 0.001$) and a 20% increase in kidney weight ($0.02 > P > 0.01$). By comparison renal function in rats treated with i.v. carboplatin or p.o. JM216 was normal. At 4 days rats treated with i.v. cisplatin showed histological changes consistent with drug-induced injury, i.e. gross dilatation of the renal tubules, medullary tubular cast formation and necrosis of the renal tubular epithelium as manifest by pyknotic change, clearing of nuclear chromatin and cellular sloughing into the tubular lumen. In contrast, the histological appearances of rat kidney following i.v. carboplatin or p.o. JM216 treatment were within normal limits. Urinary N-acetyl-β-glucosaminidase (NAG) activity, urinary leucine aminopeptidase (LAP) activity and urinary output 72 to 96 h following treatment were similar in control and treatment groups.

### Discussion

We have studied the nephrotoxicity of a series of six p.o. ammine/amine Pt(IV) dicarboxylate complexes in rodents with variations in chemical structure at the (i) axial dicarboxylate (formato, acetato, butyrato or ethylcarbamate substituents), (ii) amine (cyclopentylamine or cyclohexylamine substituents), and (iii) leaving group (dichloro of oxalato substituents) positions. These compounds have been identified as potential candidates for clinical testing as orally active Pt-based drugs (Harrap *et al.*, 1991).

These studies of nephrotoxicity in rodents included i.v. cisplatin and i.v. carboplatin as controls. The effects of i.v. cisplatin were similar to those previously described (Harrap *et al.*, 1980; Jodrell *et al.*, 1991; Ward *et al.*, 1976) in that this agent caused proteinuria, glycosuria, reduced GFR and histological kidney damage in the mouse, and elevated plasma creatinine, elevated plasma urea, reduced creatinine clearance, urinary glucose wasting, increased kidney weight and renal histological damage in the rat. By comparison, i.v. carboplatin effected neither renal function nor kidney histology in either species. These findings concur with their relative clinical nephrotoxicity since cisplatin can cause severe and irreversible damage to renal function in man (Daugaard *et al.*, 1988) while the toxicity of carboplatin is confined to the reductions in GFR after high-dose therapy (Gore *et al.*, 1987) and the possibility of cumulative reductions in GFR in some patient groups (Sleijfer *et al.*, 1989; Mason *et al.*, 1990).

In mice, single MTD treatments of six p.o. ammine/amine Pt(IV) dicarboxylates caused neither proteinuria, glycosuria nor major histological changes. Transient cortical fatty change, without necrosis, was recorded following treatment with one oral compound (JM291). GFR estimates in control mice were similar to reported values (Jodrell *et al.*, 1991) and neither p.o. JM216 nor p.o. JM221 treatment at the MTD caused reductions in this parameter. Above the MTD, p.o. JM221 caused a small reduction in GFR, possibly in keeping with reports of reductions in GFR during high-dose carboplatin therapy (Gore *et al.*, 1987). In rats, the time-course of elevated plasma urea following i.v. cisplatin treatment was consistent with previous descriptions (Ward *et al.*, 1976), however, plasma urea was normal following treatment with the oral phase I candidate (p.o. JM216) at time points ranging from 24 h to 10 days. Creatinine clearance values in control rats were similar to reference values (Altman & Dittmer, 1974) and unchanged 4 days following p.o. JM216, contrasting with the 10-fold reduction in creatinine clearance following i.v. cisplatin. Urinary glucose wasting and histological kidney damage in the rat was seen only with i.v. cisplatin and not with p.o. JM216. These findings suggest that single dose treatment at the MTD with p.o. ammine/amine Pt(IV) dicarboxylates is non-toxic to the kidney in rodents.

The renal effects of multiple dosing was studied and neither p.o. JM216, p.o. JM225, p.o. JM291, nor p.o. JM251 given as weekly doses for 4 weeks caused histological kidney damage in the mosue. Histological changes are amongst the most sensitive manifestations of cytotoxic drug-induced kidney damage in this species (Jodrell *et al.*, 1991), however, the lack of cumulative damage of p.o. ammine/amine Pt(IV) dicarboxylates studies has not been confirmed by studies of renal function to date. In mice, following single doses of i.v. cisplatin, plasma creatinine and urea were within the control range. This is consistent with previous suggestions of these being of poor utility for screening purposes because they are insensitive manifestations of Pt-induced kidney damage, both in this species (Jodrell *et al.*, 1991) and in man (Daugaard *et al.*, 1988*b*). Similarly in rats, we found neither urinary NAG nor LAP activity elevated following cisplatin treatment.

In conclusion, this series of p.o. ammine/amine Pt(IV) dicarboxylates are not toxic to the kidney in rodents at maximum tolerated doses and on this basis are suitable for development as oral Pt outpatient therapy. Their lack of nephrotoxicity was comparable to i.v. carboplatin suggesting that the co-administration of i.v. hydration, such as is necessary with i.v. cisplatin, may not be required.

This study was supported by grants to The Institute of Cancer Research: Royal Cancer Hospital from the Cancer Research Campaign, The Johnson Matthey Technology Centre and Bristol-Myers Squibb (UK). The Royal Marsden Hospital Pathology Laboratories kindly prepared the histological specimens and analysed the plasma samples.

Abbreviations: cisplatin, *cis*-diamminedichloroplatinum(II); carboplatin, *cis*-diamminecyclobutanedicarboxylatoplatinum(II); GFR, glomerular filtration rate; h, hour; i.v., intravenously administered; p.o., orally administered; MTD, maximum tolerated dose; Pt, platinum.

### References

AL-SARRAF, M., FLETCHER, W., OISHI, N., PUGH, R., HEWLETT, J.S., BALDUCCI, L., MCCRACKEN, J. & PADILLA, F. (1982). Cisplatin hydration with and without mannitol diuresis in refractory disseminated melanoma: A Southwest Oncology Group Study. *Cancer Treat. Rep.*, **66**, 31–35.

ALTMAN, P.L. & DITTMER, D.S. (1974) (eds). *Biology Data Book Volume III, (2nd ed.)*. Federation of American Societies of Experimental Biology, Bethesda, Maryland, pp. 2004.

CALVERT, A.H., HARLAND, S.J., NEWELL, D.R., SIDDIK, Z.H., JONES, A.C., MCELWAIN, T.J., RAJU, S., WILTSHAW, E., SMITH, I.E., BAKER, J.M., PECKHAM, M.J. & HARRAP, K.R. (1982). Early clinical studies with *cis*-diammine-1,1-cyclobutane dicarboxylate platinum (II). *Cancer Chemother. Pharmacol.*, **9**, 140–147.

DAUGAARD, G., ABILDGAARD, U., HOLSTEIN-RATHLOU, N.-H., BRUUNSHUUS, I., BUCHER, D. & LEYSSAC, P.P. (1988*a*). Renal tubular function in patients treated with high-dose cisplatin. *Clin. Pharmacol. Ther.*, **44**, 164–172.

DAUGAARD, G., ROSSING, N. & RORTH, M. (1988*b*). Effects of cisplatin on different measures of glomerular function in the human kidney with special emphasis on high-dose. *Cancer Chemother. Pharmacol.*, **21**, 163–167.

DECONTI, R.C., TOFTNESS, B.R., LANGE, R.C. & CREASEY, W.C. (1973). Clinical and pharmacological studies with *cis*-diamminedichloroplatinum(II). *Cancer Res.*, **33**, 1310–1315.

GOLDSTEIN, R.S., NOORDEWEIR, B., BOND, J.T., HOOK, J.B. & MAYOR, G.H. (1981). *Cis*-dichlorodiammineplatinum nephrotoxicity: time-course and dose response of renal functional impairment. *Toxicol. Appl. Pharmacol.*, **60**, 163–175.

GONZALEZ-VITALE, J.C., HAYES, D.M., CVITKOVIC, E. & STERNBERG, S.S. (1977). The renal pathology in clinical trials of *cis*-platinum (II) diamminedichloride. *Cancer*, **39**, 1362–1371.

GORE, M.E., CALVERT, A.H. & SMITH, I.E. (1987). High dose carboplatin in the treatment of lung cancer and mesothelioma: a phase I dose escalation study. *Eur. J. Cancer Clin. Oncol.*, **23**, 1391–1397.

HARRAP, K.R., JONES, M., WILKINSON, C.R., CLINK, H.M., SPARROW, S., MITCHLEY, B.C.V., CLARKE, S. & VEASEY, A. (1980). Antitumour, toxic and biochemical properties of cisplatin and eight other platinum complexes. In Prestayko, A.W., Crooke, S.T., Carter, S.K. (eds), *Cisplatin Current Status and New Developments*, pp. 193–212. Academic Press: New York.

HARRAP, K.R., MURRER, B.A., GIANDOMENICO, C., MORGAN, S.E., KELLAND, L.R., JONES, M., GODDARD, P.M. & SCHURIG, J. (1991). Ammine/amine platinum(IV) dicarboxylates: a novel class of complexes which circumvent intrinsic cisplatin resistance. In *Platinum and Other Metal Coordination Compounds in Cancer Chemotherapy*, Howell, S.B. (ed.) pp. 391–399. Plenum Press: New York.

JODRELL, D.I., NEWELL, D.R., MORGAN, S.E., CLINTON, S., BENSTED, J.P.M., HUGHES, L.R. & CALVERT, A.H. (1991). The renal effects of $N^{10}$-propargyl-5,8-dideazafolic acid (CB3717) and a non-nephrotoxic analogue ICI D1694, in mice. *Br. J. Cancer*, **64**, 833–838.

JONES, B.R., BHALLA, R.B., MLADEK, J., KALEYA, R.N., GRALLA, R.J., ALCOCK, N.W., SCHWARTZ, M.K., YOUNG, C.W. & REIDENBERG, M.M. (1980). Comparison of methods of evaluating nephrotoxicity of cis-platinum. *Clin. Pharmacol. Ther.*, **27**, 557-562.

KELLAND, K.R., MURRER, B.A., ABEL, G., GIANDOMENICO, C.M., MISTRY, P. & HARRAP, K.R. (1992). Ammine/amine platinum (IV) dicarboxylates: a novel class of platinum complex exhibiting selective cytotoxicity to intrinsically cisplatin resistant human ovarian carcinoma cell lines. *Cancer Res.*, **52**, 822.

LOEHRER, P.J. & EINHORN, L.H. (1984). Cisplatin. *Ann. Intern. Med.*, **100**, 704-713.

MASON, M.D., NICHOLLS, J. & HORWICH, A. (1990). The effect of carboplatin on renal function in patients with metastatic germ cell tumours. *Br. J. Cancer*, **63**, 630-633.

ROSSOF, A.H., SLAYTON, R.E. & PERLIA, C.P. (1972). Preliminary clinical experience with *cis*-diamminedichloroplatinum (II) (NSC 119875, CACP). *Cancer*, **30**, 1451-1456.

SIDDIK, Z.H., DIBLE, S.E., BOXALL, F.E. & HARRAP, K.R. (1986). Renal pharmacokinetics and toxicity of cisplatin and carboplatin in animals. In *Biochemical Mechanisms of Platinum Antitumour Drugs*, McBrien, D.C.H. & Slater, T.F. (eds), pp. 171-198. IRL Press Ltd: Oxford, England.

SLEIJFER, D.TH., SMIT, E.F., MEIJER, S., MULDER, N.H. & POSTMUS, P.E. (1989). Acute and cumulative effects of carboplatin on renal function. *Br. J. Cancer*, **60**, 116-120.

TALLARIDA, R.J. & MURRAY, R.B. (1987). *Manual of Pharmacologic Calculations with Computer Programs (2nd ed.)*. Springer-Verlag: New York.

WARD, J.M., YOUNG, D.M., FAUVIE, K.A., WOLPERT, M.K., DAVIES, R. & GUARINO, A.M. (1976). Comparitive nephrotoxicity of platinum cancer chemotherapeutic agents. *Cancer Treatment Rep.*, **60**, 1675-1678.